# Parathyroid carcinoma during pregnancy: a novel pathogenic CDC73 mutation – a case report

**DOI:** 10.1186/s12902-022-01169-2

**Published:** 2022-10-25

**Authors:** Chinthana Dematapitiya, Chiara Perera, Sivatharshiya Pathmanathan, Vindya Subasinghe, Gayani Anandagoda, Vajira Dissanayaka, Umesha Wijenayake, Preethi Dissanayake, Kavinga Gamage, Piyumi Wijewickrama, Manilka Sumanatilleke

**Affiliations:** 1 National Hospital of Sri Lanka, Colombo, Sri Lanka; 2 Provincial Directorate of Health Services-Western Province, Colombo, Sri Lanka; 3 Faculty of Medicine, University of Colombo, Colombo, Sri Lanka

**Keywords:** Parathyroid carcinoma, CDC73 mutation, Case report, Pregnancy, PTH-dependent hypercalcemia

## Abstract

**Background:**

Parathyroid carcinoma is an uncommon cause of PTH-dependent hypercalcemia. Only a handful of cases have been reported of parathyroid carcinoma during pregnancy.

**Case presentation:**

Twenty-four – Year – old female presented with proximal myopathy was found to have hypercalcemia. Her serum corrected total calcium was – 15 mg/dl (8.5 – 10.3), serum phosphate – 2.3 mg/dl (2.5 – 4.5), intact PTH – 118 pg/ml (20 – 80), Vitamin D – 15 ng/ml and Urine Ca/Cr ratio – 2.1 (0.1 – 0.2). Her CECT–neck revealed a well-defined mass lesion posterior to the right lobe of the thyroid – 2.6 cm × 2.5 cm × 2.9 cm in size. She was started on vitamin D supplementation, and she underwent right lower focal parathyroidectomy. Her PTH levels normalized following surgery. Her histology revealed an atypical parathyroid adenoma. She was treated with calcium and vitamin D. Her follow up was uneventful.

One year following initial surgery the patient became pregnant and at 16 weeks of POA, the patient presented with a rapidly enhancing neck mass for one week duration. Her biochemical investigations were suggestive of a recurrence of primary hyperparathyroidism. Her ultrasound scan of the neck revealed a well-defined discreate hypoechoic nodule, superior to the thyroid isthmus which was confirmed by a non-contrast MRI scan of the neck. She underwent an uncomplicated second trimester parathyroid tumour excision with normalization of post op PTH. Her histology revealed a parathyroid carcinoma with vascular and capsular invasion. Her genetic studies revealed a novel frameshift mutation of the CDC73 gene. She was treated with calcium and vitamin D supplementation and closely followed up with ionized calcium and PTH levels which were normal throughout the pregnancy. She had an uncomplicated caesarean section at a POA of 37 weeks. Currently she is twelve weeks post-partum, in remission of disease.

**Conclusion:**

This case shows the importance of stringent follow up of atypical parathyroid adenoma patients, the benefit of second trimester surgery in management of hypercalcemia due to parathyroid carcinoma during pregnancy and the importance of identifying the novel CDC73 gene mutation.

## Background

Primary hyperparathyroidism (PHPT) is defined as hypercalcemia with inappropriately elevated parathyroid hormone (PTH) levels for the hypercalcaemic state due to abnormal, incompletely regulated secretion of PTH from one or more of the four parathyroid glands [1]. In a pregnant woman with PHPT, parathyroid adenoma was found in 89%, parathyroid hyperplasia in 8% and parathyroid carcinoma in 2% of patients [2].

Parathyroid carcinoma (PC) can occur either sporadically or as part of a familial syndrome. Familial syndromes, such as hyperparathyroidism-jaw tumour (HPT-JT) syndrome, multiple endocrine neoplasia (MEN) types 1 and 2A and familial isolated hyperparathyroidism have been linked with parathyroid carcinoma [3]. Inactivation mutations of HRPT2 (CDC73 or Parafibromin), retinoblastoma (RB) and p53 genes [4] and activation mutation of cyclin D1 gene could be involved in the pathogenesis of parathyroid carcinoma [5].

CDC73 variants were identified in 12.4% of referred PHPT patients, with HPT-JT syndrome in 3.3%, familiar isolated PHPT in 5.6%, apparently sporadic PC in 2.2% and apparently sporadic parathyroid adenoma in 1.1%. Tumour suppressor protein called parafibromin is encoded by CDC 73 gene. Most of the pathogenic CDC73 variants are frameshift and nonsense variants, but missense variants as well as insertions and deletions have been reported [6].

Our case is unique, mainly with regards to two aspects. First aspect being only 8 cases have been reported in literature regarding parathyroid carcinoma in pregnancy [7–14] and secondly, identified frameshift mutation of *CDC73* gene is a novel variant.

## Case presentation

A 24- year-old previously healthy female was referred to the neurology clinic due to difficulty in walking for a duration of one month. Further clinical evaluation revealed proximal muscle weakness. Her electromyography confirmed the proximal myopathy and her corrected serum calcium was 15 mg/dl (8.5 – 10.3). Due to hypercalcemia, she was referred to the Endocrinology department for further management.

**Fig. 1:**
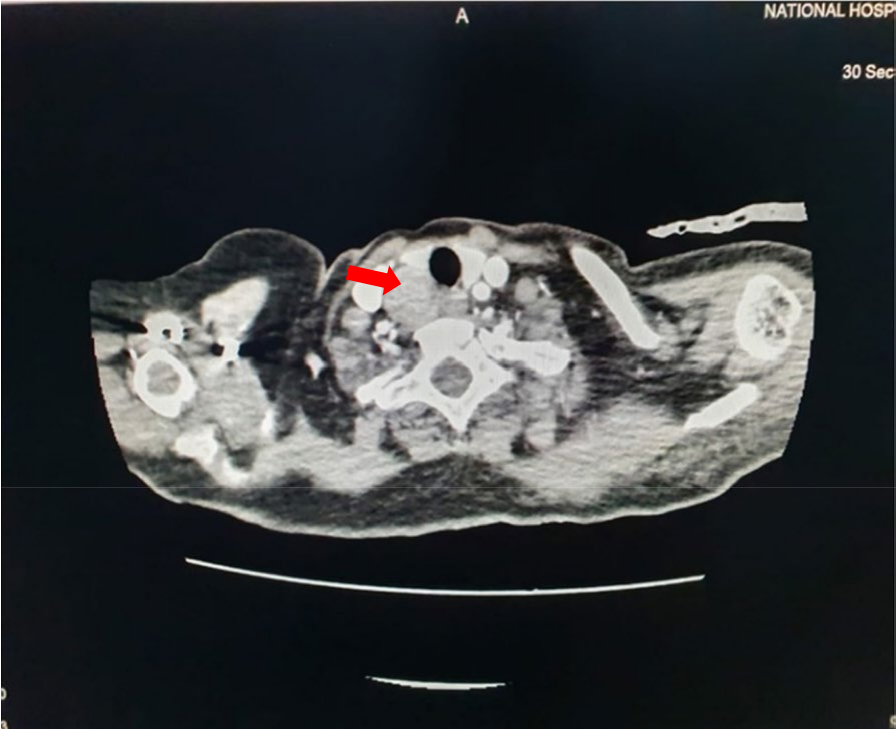
CECT-neck showing right superior parathyroid adenoma

**Fig. 2:**
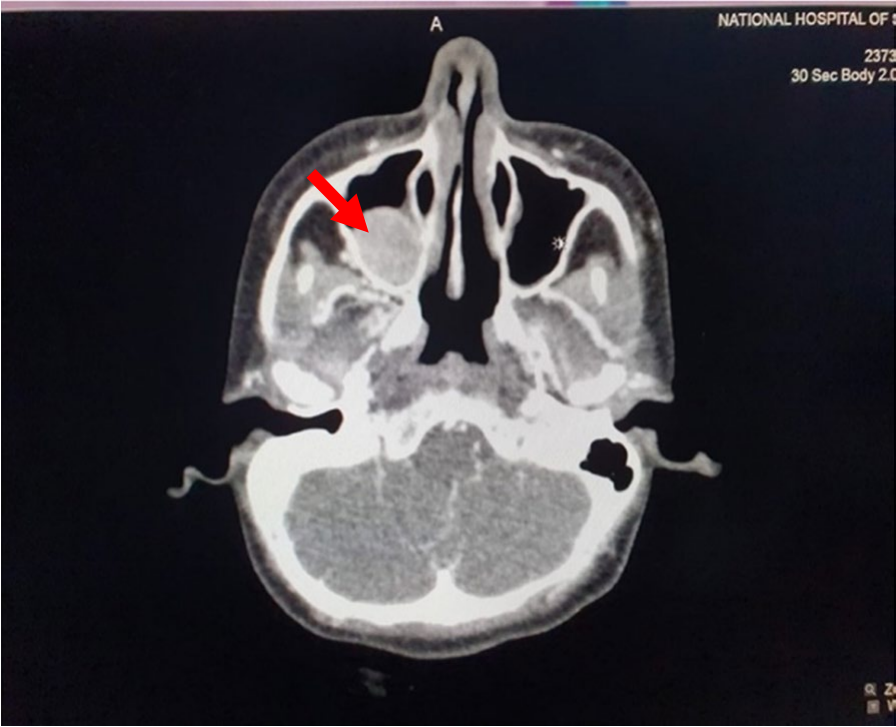
CECT-para nasal sinuses showing an enhancing lesion in the right maxillary sinus

The patient was evaluated for hypercalcemia in the Endocrinology clinic. There was a positive family history of hypercalcemia where her mother was also found to have hypercalcemia. Her initial biochemical evaluation revealed a serum corrected total calcium – 15 mg/dl (8.5 – 10.3), serum phosphate – 2.3 mg/dl (2.5 – 4.5), intact PTH – 118 pg/ml (20 – 80), vitamin D – 15 ng/ml and spot urine calcium to creatinine ratio (spot urine ca/cr) – 2.1 (0.1 – 0.2) without imaging evidence of nephrolithiasis. Dual energy x ray absorptiometry (DEXA) scan revealed osteoporosis with distal forearm Z score of – 3.2. Her Contrast Enhanced Computed Tomography (CECT) – chest, abdomen, and neck revealed a well-defined mass posterior to the right lobe of the thyroid, measuring 2.6 cm × 2.5 cm × 2.9 cm in size suggestive of a right parathyroid adenoma (Fig. 1) and an enhancing lesion in the right maxillary sinus measuring 1.9 cm × 2.3 cm in size (Fig. 2). Following these investigations, a presumptive diagnosis of primary hyperparathyroidism due to the right parathyroid adenoma was made, and she underwent a right focal parathyroidectomy following vitamin D supplementation.

Her pre-incision PTH was 3700 pg/ml with vitamin D levels of 35 ng/ml and post-incision PTH was 30 pg/ml. Her histology revealed a right parathyroid tumour with no definitive vascular invasion with a high mitotic count (4/10 HPF) and high Ki index of 12% but capsular invasion was difficult to comment suggestive of an atypical parathyroid adenoma There was no evidence of fibrous bands, necrosis, or extension to peri-glandular tissue. Resection margin was clear from the tumor and hemosiderin deposition was not identified.

Her follow up was uncomplicated until 10 months following surgery. After 10 months of surgery, she was found to be pregnant at a period of amenorrhoea (POA) of 8/52. Biochemical investigations revealed a serum corrected total calcium of 8.1/dl (8.5 – 10.3), serum phosphate – 2.2 mg/dl (2.5 – 4.5) with intact PTH – 232 pg/ml (20 – 80) and vitamin D level of 18 ng/ml. We continued vitamin D and calcium supplementation in view of normalizing the vitamin D levels.

**Fig. 3:**
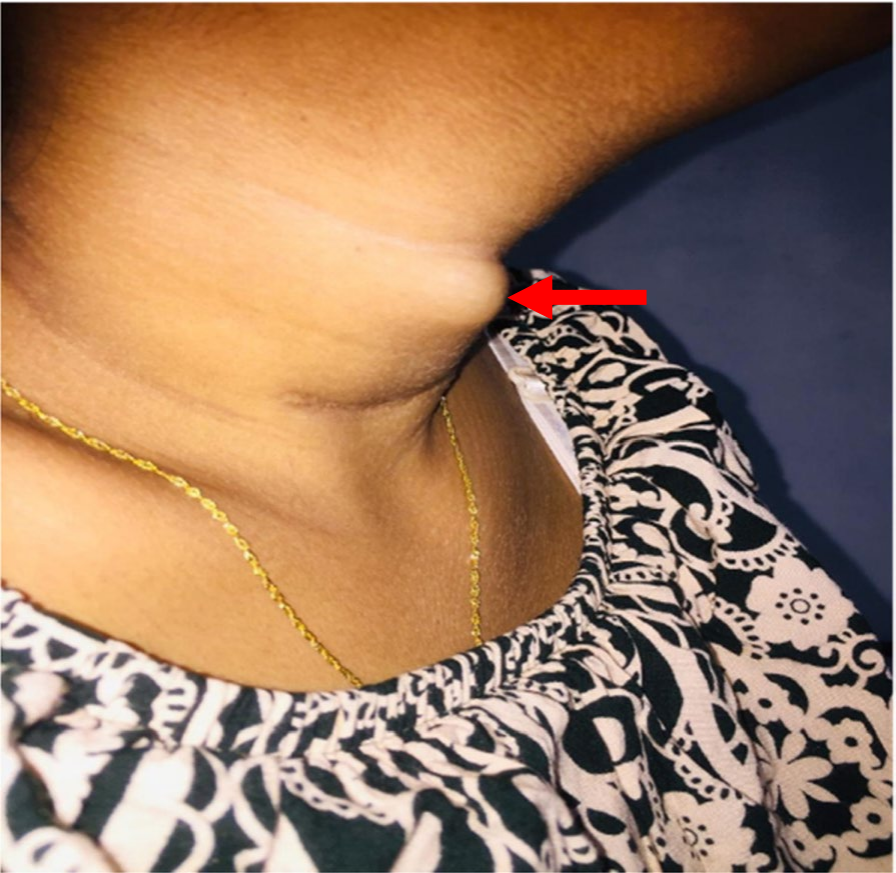
Patient presenting with rapidly progressive neck lump

After 12 months of initial surgery at a POA of 16/52, the patient presented with a rapidly progressive neck lump within a duration of 1 week (Fig. 3). Her serum corrected total calcium was 10.8 mg/dl (8.5 – 10.3), serum phosphate – 2.0 mg/dl (2.5 – 4.5), vitamin D – 45 ng/ml and intact PTH was 540 pg/ml (20 – 80). She had an elevated spot urine calcium to creatinine ratio of 1. 5, but no evidence of nephrolithiasis. Since she was pregnant DEXA scan was not done.

**Fig. 4:**
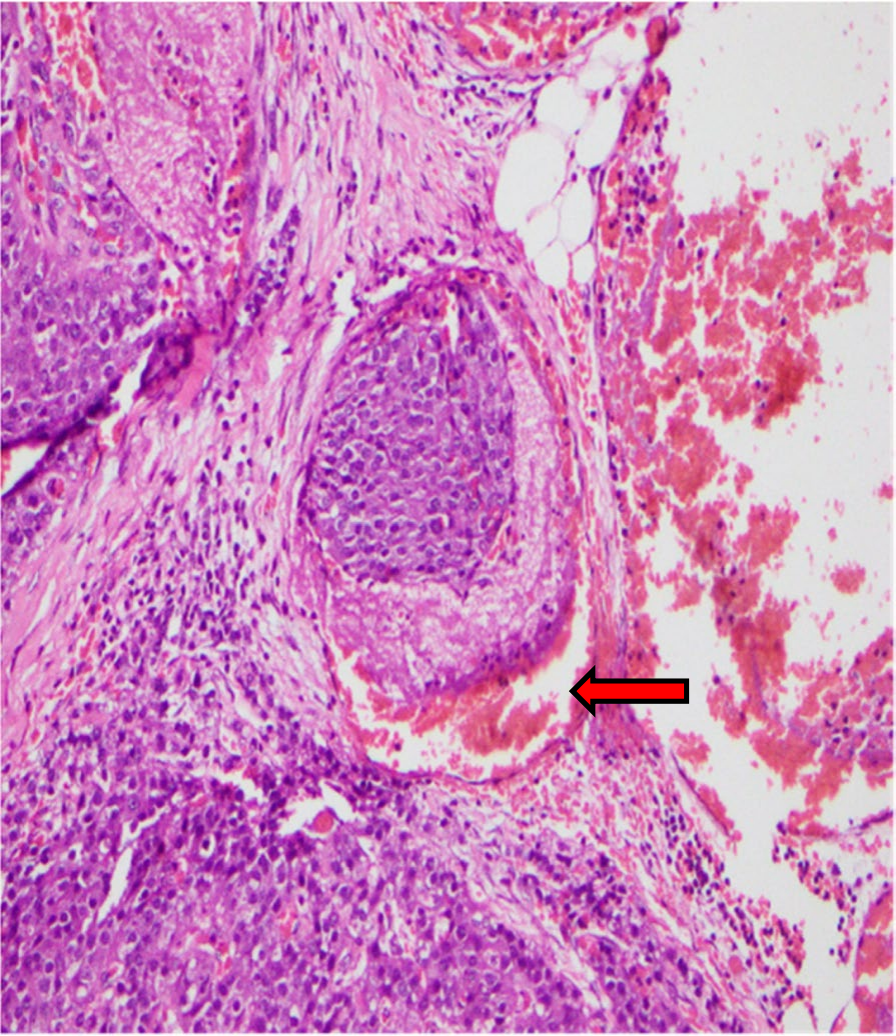
Histology of tumour with evidence of vascular invasion

An urgent Ultrasound Scan (USS) – neck revealed a well-defined discreate hypoechoic nodule superior to the thyroid isthmus. A non-contrast Magnetic Resonance Imaging (MRI) of neck /paranasal sinuses & upper chest showed a well-defined lesion in the subcutaneous tissue plane of the neck at the thyroid isthmus level, but there was no evidence of the previously noted lesion in the right maxillary sinus. She underwent a parathyroid tumour excision, and during the surgery, a 2 × 2 cm lump was noted in the midline from the trachea, up to the subplatysmal plane, but there was no connection with the thyroid gland. The left superior and inferior and right superior parathyroid glands were normal, and they were preserved. Intraoperative pre-incisional PTH was 568 pg/ml with vitamin D level of 45 ng/ml and post-incisional PTH was 68 pg/ml. Her post-operative inward stay was uncomplicated with satisfactory foetal wellbeing. She was discharged with calcium and vitamin D supplementation. Her histology revealed a tumour of 2 cm × 1.5 cm × 1.2 cm in size, encapsulated nodule composed of nests and sheets of tumor cells with round to ovel nuclei, dense chromatin pattern and inconspicuous nucleoli. Mitotic figures are observed (12/10 HPF). Atypical mitoses were not seen. Coagulative tumor necrosis, diffuse sheet like monotonous small cells or broad intra-tumoral fibrous bands were not evident. There were multiple foci of unequivocal vascular invasion at the periphery of the tumor suggestive of parathyroid carcinoma. Perineural invasion was not evident, but there was infiltration of adjacent fatty tissue with capsular invasion (Fig. 4). Closest resection margin was 1 mm away from the tumor.

**Fig. 5:**
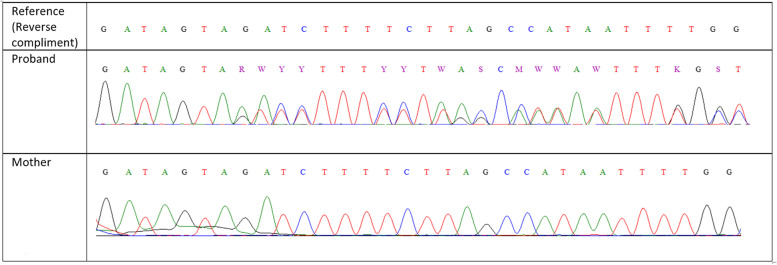
Sanger Sequence of patient and her mother

Whole exome sequencing was performed in the proband which revealed a likely pathogenic heterozygous frameshift variant in the *CDC73* gene c.584delC | p. Ser195fs (novel variant). Sanger sequencing was performed to confirm the variant identified in the proband. Genetic testing for MEN 1 gene was negative in this patient. Her mother was also found to have primary hyperparathyroidism secondary to parathyroid adenoma and underwent 4 gland exploration. Yet, her histology was suggestive of parathyroid adenoma without atypical features. She was also subjected to Sanger sequencing. However, the mother did not harbour the variant. Sanger images of both are as below (Fig. 5).

She was followed up throughout her pregnancy, both clinically and biochemically for hypercalcemia and foetal wellbeing. Her pregnancy was uncomplicated after the second trimester parathyroid surgery, and she underwent an uncomplicated caesarean section at a POA of 37 weeks. She delivered a healthy baby with a birth weight of 2.7 kg and the baby did not develop neonatal hypocalcaemia. Following 3 months of delivery her corrected calcium was 8.9 mg/dl with intact PTH – 68 ng/dl. Currently following 6 months of her delivery corrected calcium is 8.7 mg/dl with intact PTH – 65 ng/dl.

## Discussion and conclusions

Parathyroid carcinoma (PC) is usually diagnosed in the fifth decade of life and has an equal frequency of occurrence in both sexes [15]. But since our patient presented in her second decade, we had to strongly suspect a familial syndrome causing PC. Hyperparathyroidism jaw tumor syndrome is an autosomal dominant disease which is known to cause parathyroid carcinoma. It consists of mutations in the CDC 73 gene, primary hyperparathyroidism due to parathyroid tumours, ossifying fibroma (usually in the maxilla or mandible) and a variety of uterine and renal abnormalities. We suspect this familial syndrome in our patient because she had a CDC 73 gene mutation, parathyroid carcinoma and possible ossifying fibroma in the right maxillary sinus, but no evidence of uterine or renal abnormalities. Unfortunately, we couldn’t biopsy the right maxillary lesion to confirm the ossifying fibroma [15].

There are several clinical, diagnostic, and therapeutic problems with parathyroid carcinoma when it occurs in pregnancy. A palpable rapidly enlarging neck mass (30 – 70%) and recurrent laryngeal nerve palsy are important clinical signs which suggest PC rather than benign hyperparathyroidism [16]. During pregnancy our patient presented with a rapidly enlarging neck lump suggestive of parathyroid carcinoma. PHPT in pregnancy could be linked with adverse complications. Analysis of outcome of 40 pregnant patients with PHPT, there was a 27.5% incidence of foetal deaths and a neonatal tetany incidence of 19% [17]. Major maternal complications identified were nephrolithiasis, acute pancreatitis, and bone disease.

Fibrous bands with trabecular architecture, capsular and vascular invasion and a high mitotic index in the parathyroid histology are suggestive of PC. Some have used atypical adenoma for lesions which has PC features but questionable invasion. But with the use of immunohistochemistry, now parathyroid adenoma and carcinoma can be clearly differentiated. The main immunohistochemistry features suggestive of PC are, loss of expression of parafibromin, Rb, p27, Bcl-2a, mdm -2 and APC with the positivity of galectin and overexpression of P53 and Ki index of > 5% [4]. Our patient, following initial surgery had a high mitotic index, questionable invasion, overexpression of P53 with Ki index of 12 are more suggestive of parathyroid carcinoma rather than atypical adenoma. Parafibromin staining is very important for immunohistopathological diagnosis of PC, as CDC – 73 gene is responsible for expression of parafibromin protein. In our patient, staining of parafibromin and B catenin proteins were not done due to its unavailability.

In our patient, the Whole exome sequencing was done in blood, and not in parathyroid tissue, and it was performed by Illumina® NovaSeq® 6000 Next Generation Sequencer using the SureSelectXT ® Human (Mouse) All Exon V6 5190–8864 kit. With the in-house bioinformatics pipeline, the genetic analysis was performed. Mapping the paired end sequences to GrCh37 human reference genome and variant calling was done using BWA-mem algorithm and Genome Analysis Tool Kit (GATK). Generated variant calling format file (VCF) was annotated using SNP‐eff with Refseq, population and clinical database information. A virtual gene panel was created including the genes responsible for Multiple Endocrine Neoplasia (MEN) and Hyperparathyroidism, which was then used to identify the variant associated with the phenotype and the clinical features of the proband. Benign variants were filtered out according to the standard American College of Medical Genetics (ACMG) guidelines [18].

Analysis of the remaining variants revealed a novel heterozygous single base deletion (NM_024529.5: c.584delC) in the exon 07 of the CDC73 gene which may create a frameshift (p. Ser195Leufs*7) and a premature protein truncation. This variant is predicted to be pathogenic, when analysed using the MutationTaster software [19].

Furthermore, this single base deletion causes the loss of the C- terminal domain of theparafibromin protein. Hence the interaction of the C- terminal domain of the parafibromin protein with the RNA polymerase II-associated factor 1 homolog protein (PAF1) and DNA-directed RNA polymerase II subunit RPB1(POLR2A) is lost. Moreover, the premature protein lost during the interaction with Catenin beta-1 protein, which is a key downstreamcomponent of the canonical Wnt signalling pathway [20].

When parathyroid carcinoma occurs in pregnancy, the main cause for increase maternal and foetal mortality is Hypercalcemia. Hypercalcemia can be managed medically or surgically. Both approaches have its own benefits and risks. Patient symptoms, severity of disease and age at gestation at the time of diagnosis are main factors which will decide therapeutic option. If the patient was diagnosed during the 1^st^ or 2^nd^ trimesters in pregnancy with severe hypercalcemia (Ionized calcium > 2.85 mg/dl), second trimester surgery is generally preferred over medical management [3]. According to Carella and Gossain [21] there are higher foetal complications in 3rd trimester surgery when compared to the second trimester. In our patient, we suspected an underline parathyroid carcinoma, before the second surgery. But at that time, she was pregnant at 16 weeks of POA. Even though en bloc resection with thyroidectomy was considered, we had to consider the duration of surgery and the harmful effects of prolong anesthesia to the fetus as well as mother, as en block resection will take a considerable time, compared to focal removal of the parathyroid mass. Therefore, after discussing the benefits and risks with the patient, we decided to go ahead with focal removal of the parathyroid mass.

Medical management is mainly indicated in mild hypercalcemia, and it consist of volume expansion, loop diuretics, bisphosphonates, denosumab, calcimimetics and calcitriol. But in pregnancy, most of the above are contraindicated. But calcimimetics and calcitriol have been used successfully without any foetal mortality. However, they have not shown to alter the natural progression of the disease, and data on long term efficacy of calcimimetics are lacking [22].

Parathyroid carcinoma has a high recurrence rate (23 – 51%). So, patients with parathyroid carcinoma should be followed up throughout their lifetime with serum calcium and intact PTH, every 6 months for the initial 5 years following surgery, and then annually. They should also undergo ultrasound scans of the neck annually with biochemical monitoring as above.

This case shows the importance of stringent follow up with atypical parathyroid adenoma patients, benefit of second trimester surgery in management of hypercalcemia due to parathyroid carcinoma during pregnancy and importance of identifying the novel mutations of CDC73 gene.

## Data Availability

Data to support the findings of this study is available from the corresponding author upon reasonable request. Data is available at SRA NCBI. Accession to cite for these SRA data: PRJNA880506. Submission ID: SUB12053939. SRA records will be accessible with the following link: https://www.ncbi.nlm.nih.gov/sra/PRJNA880506

## Abbreviations

PTH: Parathyroid Hormone
Ca/Cr: Calcium/Creatinine
CECT: Contrast Enhanced Computed Tomography
MRI: Magnetic Resonance Imaging
POA: Period of Amenorrhoea
PHPT: Primary hyperparathyroidism
RB: Retinoblastoma
HPT-JT: Hyperparathyroidism-Jaw Tumour
HPF: High Power Field
USS: Ultra Sound Scan
PC: Parathyroid Carcinoma
MEN: Multiple Endocrine Neoplasia
ACMG: American College of Medical Genetics
RNA: Ribonucleic Acid
DNA: Deoxyribonucleic Acid
DEXA: Dual Energy X-ray Absorptiometry
